# Climate and Soil Type Together Explain the Distribution of Microendemic Species in a Biodiversity Hotspot

**DOI:** 10.1371/journal.pone.0080811

**Published:** 2013-12-18

**Authors:** Romain Nattier, Philippe Grandcolas, Roseli Pellens, Hervé Jourdan, Arnaud Couloux, Simon Poulain, Tony Robillard

**Affiliations:** 1 Muséum national d’Histoire naturelle, Département Systématique et Evolution, Paris, France; 2 Laboratoire Evolution, Génomes et Spéciation, UPR 9034 CNRS, Gif sur Yvette, France and Université Paris Diderot, Sorbonne Paris Cité, France; 3 Institut Méditerranéen de Biodiversité et d’Ecologie terrestre et marine, Aix-Marseille Université/CNRS/IRD/UAPV, UMR 237 IRD, Centre IRD de Nouméa, Nouméa, Nouvelle-Calédonie; 4 Genoscope, Centre national de Séquençage, Evry, France; Institute of Biochemistry and Biology, Germany

## Abstract

The grasshopper genus *Caledonula,* endemic to New Caledonia, was studied to understand the evolution of species distributions in relation to climate and soil types. Based on a comprehensive sampling of 80 locations throughout the island, the genus was represented by five species, four of which are new to science, of which three are described here. All the species have limited distributions in New Caledonia. Bioclimatic niche modelling shows that all the species were found in association with a wet climate and reduced seasonality, explaining their restriction to the southern half of the island. The results suggest that the genus was ancestrally constrained by seasonality. A molecular phylogeny was reconstructed using two mitochondrial and two nuclear markers. The partially resolved tree showed monophyly of the species found on metalliferous soils, and molecular dating indicated a rather recent origin for the genus. Adaptation to metalliferous soils is suggested by both morphological changes and radiation on these soils. The genus *Caledonula* is therefore a good model to understand the origin of microendemism in the context of recent and mixed influences of climate and soil type.

## Introduction

New Caledonia, located in the south-west Pacific, is considered one of the major hotspots of biodiversity [1]. The main island of the archipelago, Grande Terre, is very old and its geological basement separated from Australia 80 Ma ago, hence it has often been considered a Gondwanan refuge [2], [3], [4], [5], [6]. However, recent phylogenetic studies, in concordance with all geological studies, showed that the diversification of the fauna and flora in New Caledonia is much more recent than expected [7], [8], [9] and started 37 Mya after long episodes of total submergence in the Palaeocene and in the Eocene [10], [11], [12], [13], [14], [15].

One major feature of New Caledonian biodiversity is its strong endemism [8], [16], [17]. Regional endemism, including relicts, is extremely high, since many species or even whole groups are only found on Grande Terre [5]. Local microendemism is even more striking, with thousands of species of vertebrates, plants, molluscs and insects that have very narrow distributions limited to just one mountain or one river (e.g., snails, [18]; plants, [19]; geckos, [20]; cockroaches, [7], [21]; crickets, [22]). Recently, microendemism was found to be a dynamic feature, resulting from very recent diversification through allopatric speciation and evolving later towards less restricted distributions [23].

Such repeated speciation events giving rise to microendemism have often been assumed to be favoured by two environmental factors (review in [8]): i) the diversity of soils, especially the presence of metalliferous soils in New Caledonia that might have led to adaptive speciation [4], [24], [25], [26]; and ii) climatic variation coupled with orography, which may have determined mountaintop endemics [27] or more commonly allopatric speciation with niche conservatism on neighbouring mountains [21]. Both kinds of events are presumed to be ancient. Adaptive speciation on metalliferous soils is thought to have occurred widely when these soils covered most of the island after its emersion [28]. In the case of endemism and species richness, these are usually seen as being so important that they could only result from a long accumulation of events driven by old orogenesis and repeated climatic changes [10].

**Figure 1:**
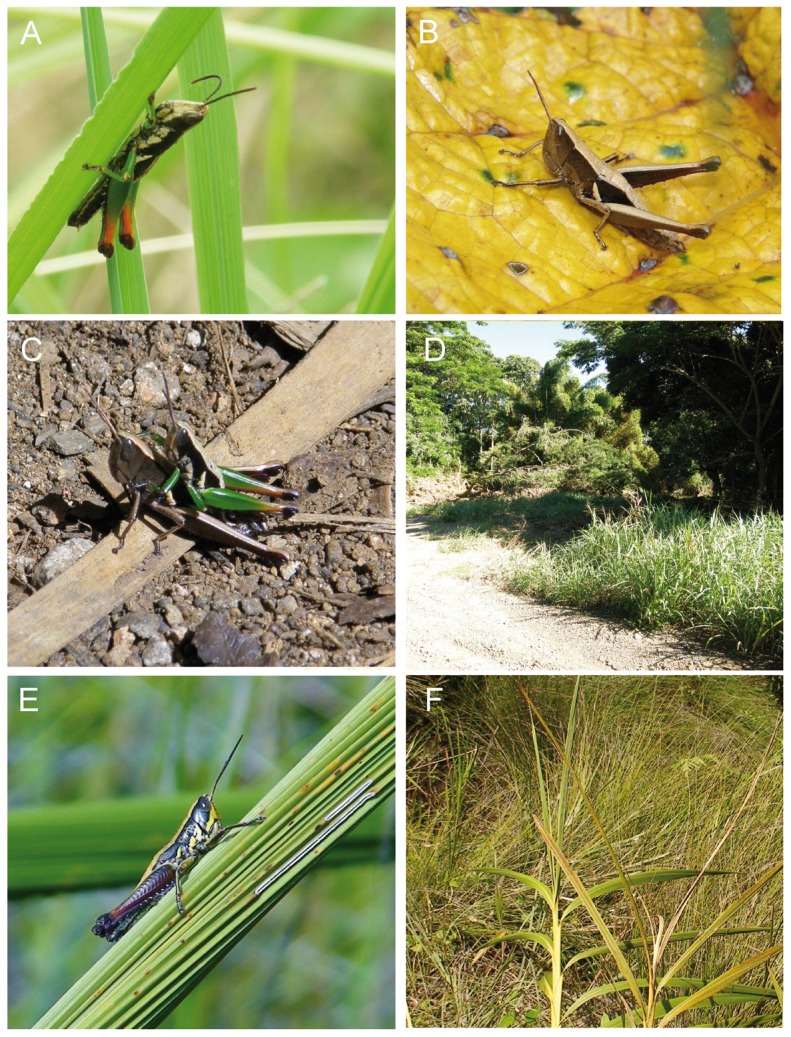
*Caledonula* species and habitat. *Caledonula fuscovittata* (A-D): male (A), female (B); couple mating in Monts Koghis during day (C), view of habitat in Col d’Amieu (D); *Caledonula amedegnatae* (E-F): male (E), view of habitat in Forêt Nord (F) (photos by T. Robillard).

Here, we study the respective contributions of these factors on the origin of species distributions and levels of endemism by investigating the diversification of the endemic grasshopper genus *Caledonula* Uvarov, 1939 (Figure 1). Grasshoppers of this genus inhabit open herbaceous habitats close to forest edges, which give them more potential abilities to disperse than in forest habitat, and they feed on leaves of various Poaceae. They combine several features that allow us to test the influence of climate and soil diversity. The distribution of the genus is restricted to the southern half of the island, suggesting that environmental factors may play a constraining role. The different species show contrasting distribution areas, corresponding to different levels of microendemism: One species is largely distributed in most of the southern part of the island, while the others are found only in very limited areas. Finally, some species only occur on metalliferous soils and some only on non-metalliferous soils, and these have morphological differences related to feeding behaviour.

Here we try in particular to determine why present distributions are highly restricted in this group. Accordingly, we studied the origin of geographical distributions of *Caledonula* species in the light of bioclimatic niche modelling, orography and soil distribution, with reference to a dated molecular phylogeny.

## Materials and Methods

### 2.1 Sampling

**2.1.1. Taxonomic sampling.** The study was mostly based on specimens collected by sight in the field in 2008–2009 (ANR BIONEOCAL), complemented by specimens from the following natural history collections: Muséum national d’Histoire naturelle, Paris, France (MNHN); Institut Agronomique de Nouvelle-Calédonie, station fruitière de Pocquereux, La Foa, Nouvelle-Calédonie (IAC); Bernice P. Bishop Museum, Honolulu, Hawaii, USA (BPBM); and the Natural History Museum, London, UK (BMNH). Museum specimens from the last three institutions were loaned to MNHN where the study was performed (see lists of studied materials for details of specimens per institution). Newly collected materials were obtained under a permit for field collection from the Directions de l’Environnement, Province Sud and Province Nord of New Caledonia. Additional information came from Kaltenbach [29], [30].

Taxonomic sampling was carried out at 80 localities throughout Grande Terre, Ile des Pins and Loyalty Islands. We gathered 153 specimens, representing 4 species (including 3 new species), at 17 localities from the centre-east (Houailou) to the south-east (Forêt Nord) (Table 1). Most of the sampled sites were published in [7], [21], [22], [23], [31], [32], [33], [34], but some of them are new. We present these data in the results section to indicate the distribution pattern of *Caledonula*.

**Table 1:** Taxonomic information, locality, altitude and number of specimens included in the study.

Species	Locality			Altitude	Number of specimens
***C. fuscovittata***	Mont Mou	22°04'28.5''S	166°19'50.7''E	360 m	8
		22°04'30''S	165°19'52''E	390 m	
	Basis of Mont Do	21°45'28.71''S	166°00'00.51''E	930 m	1
	Yahoué	22°12'33,38''S	166°29'17,02''E	25 m	3
	Mont Rembai	21°35'59,46''S	165°50'59,70''E	595 m	6
	Bourail	21°35'59,28''S	165°29'33,05''E	10 m	1
	Sarraméa	21°38'28,00''S	165°50'45,28''E	80 m	1
	Valley of Saint-Louis	22°13'34,05''S	166°33'00,25''E	15 m	3
	Near Bouloupari	21°50'00,44''S	166°03'44,07''E	30 m	2
	Monts Koghis	22°10'44''S	166°30'31''E	500 m	44
	Houailou	21°09'49,24''S	165°29'25,79''E	45 m	3
	Canala	21°31'60" S	165°57'0" E	105 m	3
	Col d'Amieu	21°35'17''S	165°47'56''E	460 m	49
		21°34'29''S	165°47'24''E		
		21°36'52''S	165°47'45''E	430 m	
		21°35'26''S	165°47'45''E	420 m	
		21°33'28''S	165°46'01''E	690 m	
		21°34'24''S	165°47'03''E	450 m	
		21°33'52''S	166°46'07''E	515 m	
		21°37S	165°49E	345 m	
***C. humboldti*** ** n. sp.**	Mont Humboldt	21°52'57.4''S	166°24'45.3''E	1343 m	15
		21°52'48.6''S	166°25'14.0''E	1564 m	
		21°52'50.4''S	166°25'08.5''E	1510 m	
		S 21.88277	E 166.4125	1350 m	
	Monts Dzumac	22°03'18.9''S	166°26'55.7''E	474 m	3
		22°01'09.9''S	166°27'52.5''E	878 m	
***C. grandgousieri n. sp.***	Haute Rivière Bleue	22°05'13,1"S	166°38'01,3"E	290 m	1
	Massif du Kouakoué	21°57'25.69''S	166°32'17.40''E	1280 m	1
***C. amedegnatae*** ** n. sp.**	Forêt Nord	22°19'03.5''S	166°54'58.7''E	335 m	7
		22°18'58.7''S	166°55'13.6''E	400 m	
		22°18'59.2''S	166°55'14.4''E	438 m	
	Haute Rivière Bleue	22°05'13,1"S	166°38'01,3"E	290 m	1
***Caledonula*** ** sp. ** ***(juvenile)***	Mont Mou	22°03'52.5''S	166°20'34.1''E	1105 m	1
***Caledonula*** ** sp. ** ***(juvenile)***	Haute Rivière Bleue	22°05'13.1"S	166°38'01.3"E	270 m	1

**2.1.2. Molecular sampling.** Only a few molecular phylogenies of grasshoppers at the genus level have been published since 1998 [35], [36], [37], [38], [39], [40], [41], [42], [43], [44]. We chose molecular markers according to these studies and we designed new primers for nuclear markers following studies on the Ensifera dealing with this taxonomic level (e.g., [45], [46], [47]).

Molecular sampling included sequences from 3 fragments of coding mitochondrial genes (Cytochrome b (Cytb, 346 bp), Cytochrome c Oxidase 1 (CO1, 670 bp) and Cytochrome c Oxidase 2 (CO2, 381 bp)) and 4 fragments of nuclear genes ((Elongation factor*-*1 alpha (EF1a, 367 bp), Histone Class 3 (H3, 331 bp), RNA of large ribosomal subunit (28S, 1100 bp), and Internal Transcribed Spacer 1 (ITS1, 350 bp)). The CO1 sequences showed full features of pseudogenes (high variability and many stop codons) and were thus discarded before phylogenetic analysis [48]. Two markers (28S and ITS1) were also discarded because they showed no variation (0% for ∼1450 bases, see Table S1 for GenBank accession numbers). All newly generated sequences were deposited in GenBank and the molecular samples used in the analyses are presented in Table 1. The ingroup consisted of 25 specimens, representing all *Caledonula* species. Each species is represented by specimens known from one to four localities. Three Oxyinae (*Oxya chinensis* and two Oxya spp.) and a Cyrtacanthacridinae (*Locusta migratoria*) were used as outgroups.

DNA was extracted from hind femora using the QIAamp DNA MicroKit (QIAGEN, Courtaboeuf, France) following the manufacturer’s instructions. Molecular work was carried out at the Muséum national d’Histoire naturelle (MNHN), Service de Systématique Moléculaire. The oligonucleotide primers used for polymerase chain reaction (PCR) and sequencing are listed in Table 2.

**Table 2:** Molecular sampling, voucher references and GenBank accession numbers of specimens included in the molecular study.

			Voucher specimen	GenBank accession number			
Species	Locality	Sample code	MNHN collection	Cytb	CO2	EF1a	H3
			number				
*C. fuscovittata*	Mts Koghis	Ko1	MNHN-CAELIF903	KF772354	KF772317	KF772369	KF772391
		Ko3	MNHN-CAELIF904	KF772345	KF772318	KF772386	KF772402
		Ko6	MNHN-CAELIF905	KF772346	KF772325	KF772370	KF772398
	Col d'Amieu	08P121	MNHN-CAELIF908	KF772342	KF772328	KF772366	KF772390
		08P162	MNHN-CAELIF910	KF772344	KF772316	**-**	KF772396
		08P51	MNHN-CAELIF907	KF772341	KF772338	KF772368	**-**
		08P131	MNHN-CAELIF909	KF772343	KF772324	KF772367	KF772401
		08P142	MNHN-CAELIF906	**-**	KF772337	KF772389	KF772397
	Mt Mou - bas	MMb1	MNHN-CAELIF900	KF772355	KF772329	KF772371	KF772392
		MMb2	MNHN-CAELIF901	KF772347	KF772330	KF772387	KF772399
		MMb3	MNHN-CAELIF902	KF772348	KF772319	KF772372	KF772400
	Base Mont Do	BMD	MNHN-CAELIF911	KF772362	KF772336	KF772373	KF772403
*C. humboldti* n. sp.	Mts Dzumac	MDz1	MNHN-CAELIF924	KF772358	KF772333	KF772381	KF772404
		MDz2	MNHN-CAELIF925	KF772352	KF772334	KF772382	KF772405
		MDz3	MNHN-CAELIF934	KF772353	KF772322	**-**	**-**
	Mt Humboldt	Hu4	MNHN-CAELIF915	KF772357	KF772331	KF772378	KF772394
		Hu5	MNHN-CAELIF916	KF772350	KF772332	KF772379	KF772407
		Hu6	MNHN-CAELIF919	KF772351	KF772321	KF772380	KF772408
*C. amedegnatae* n. sp.	Foret Nord	FN2	MNHN-CAELIF928	KF772360	KF772320	KF772374	KF772393
		FN3	MNHN-CAELIF929	KF772361	KF772326	KF772375	KF772406
		FN4	MNHN-CAELIF933	KF772349	KF772327	KF772376	**-**
*C. grandgousieri* n. sp.	Mont Kouakoué	GK	MNHN-CAELIF913	KF772356	KF772339	KF772377	**-**
	Haute-Rivière bleue	HRB2	MNHN-CAELIF912	KF772363	**-**	-	**-**
*Caledonula* sp. (juvenile)	Haut Mt Mou	MMh	MNHN-CAELIF935	**-**	KF772323	KF772388	KF772409
*Caledonula* sp. (juvenile)	Haute-Rivière bleue	RB	MNHN-CAELIF936	KF772359	KF772335	KF772383	KF772395
Oxyinae sp1	-	Oxsp1	-	KF772364	**-**	KF772384	KF772410
Oxyinae sp2	-	Oxsp2	-	KF772365	KF772340	KF772385	KF772411
*Oxya chinensis*	-	-	-	NC_010219	NC_010219	**-**	**-**
*Locusta migratoria*	-	-	-	NC_001712	NC_001712	AB583233	AF370817

Amplifications were performed in a 25 µL reaction volume with 0.4 µL of each 10 pM primer, 19.2 µL of H_2_0, 2.5 µL of buffer, 1.25 µL of dimethyl sulfoxide (DMSO), 1 µL of MIX, 0.15 µL of Taq polymerase and 1 µL of DNA. The PCR consisted of an initial denaturing step at 94°C for 4 min, 40 amplification cycles (denaturation at 94°C for 30 s, annealing at between 48 and 55°C (Table 3) for 40 s, and extension at 72°C for 40 s), and a final step at 72°C for 7 min. PCR products were checked on agarose gels and sequenced in both directions with the same primers at Genoscope (Evry, France). Sequences were cleaned, and coding sequences were translated using the invertebrate mitochondrial genetic code to check for the absence of stop codons using Sequencher v. 4.8 (GeneCodes Corporation, Ann Arbor, MI, USA). All genes were screened for potential contamination using the BlastX algorithm on GenBank.

**Table 3:** Primers used in this study.

Gene	Name	Sequencing primer (5'-3')	Reference	Annealing temperature
CO2	co2a	GGTCAAACAATTGAGTCTATTTGAAC	Contreras & Chapco (2006)	55°C
	co2e	CCACAAATTTCTGAACATTGACCA		
Cytb	427F	YTWGTWCAATGARTMTGAGG	Robillard & Desutter-Grandcolas (2006)	48°C
	800R	CCYARTTTATTAGGAATTGATCG		
EF1a	M51bF	ATTGGAACRGTGCCTGTGG	modified from Cho et al. (1995)	54°C
	M53bR	AACCATTTGCTATTTGTCCTG		
H3	HexAF	ATGGCTCGTACCAAGCAGACGGC	modified from Svenson & Whiting (2004)	58°C
	HexAR	ATATCCTTGGGCATGATGGTGAC		

### 2.2. Specimen preparation and terminology for description

The morphological terminology follows [49] for the phallic complex and [50] for external morphology. The study of morphology was carried out using a Leica MZ16 stereoscopic microscope with an ocular micrometric. Images of relevant structures were obtained with a Nikon D90 camera and a Macro Nikon lens (105 mm, f 28VR). Pictures were treated in post-production using Combine ZS Software. For drawing and studying the male genitalia, the terminalia were detached and cleared in a 10% KOH solution for 4 h, after which they were stored in glycerine.

### 2.3. Nomenclatural Acts

The electronic edition of this article conforms to the requirements of the amended International Code of Zoological Nomenclature, and hence the new names contained herein are available under that Code from the electronic edition of this article. This published work and the nomenclatural acts it contains have been registered in ZooBank, the online registration system for the ICZN. The ZooBank LSIDs (Life Science Identifiers) can be resolved and the associated information viewed through any standard web browser by appending the LSID to the prefix "http://zoobank.org/". The LSID for this publication is: urn:lsid:zoobank.org:pub:9FCFC50C-7049-4062-B16A-0573D616845F. The electronic edition of this work has been published in a journal with an ISSN, and it has been archived and is available from the following digital repositories: PubMed Central, LOCKSS.

### 2.4. Phylogenetic analysis

DNA sequences were aligned under Muscle 3.8.31 [51] using default parameters.

Parsimony analyses were performed under TNT [52]. The search strategy consisted first of 1000 replications of Random Addition Sequence and Tree Bisection and Reconnection (TBR). Then, to avoid local optima [53], we added 100 iterations of treefusing [54], each iteration being swapped with TBR and Subtree Pruning and Regrafting (SPR), and 20 iterations of ratchet [55], weighting characters with a factor of four. TNT was used to calculate Bootstrap (BS; [56]) and Jackknife (JS; [57]) support values with 1000 replicates.

To conduct Bayesian analyses, the substitution model of evolution was estimated using jMODELTEST v 0.1.1 [58], and the Akaike information criteria (AIC; [59], [60]) was used to select the GTR+I+G model. Analyses were performed with MrBayes 3.1.2 [61]. Four Markov chains were run simultaneously for 20 million generations, sampling every 100 generations to ensure independence of samples. The first 20,000 trees generated were determined empirically from the log-likelihood values using TRACER V1.4 and discarded as burn-in. [62]. The remaining trees were used to construct 50% majority-rule consensus trees. Two independent runs were performed to check whether convergence on the same posterior distribution was reached and whether the final trees converged on the same topology. The statistical confidence of each node was evaluated by posterior probabilities.

Since pseudogenes are known to be prevalent in grasshoppers [48], [63], [64], we conducted separate analyses for each data set and for different subsets of data to estimate the informative content of each kind of data and to check for problematic amplifications.

### 2.5. Molecular dating analysis

To minimize the effect of increased mutation rates at the intraspecific level in dating methods [65], [66], only one specimen per species was used for the dating analysis.

Because no calibration points were available (either fossil, paleogeographic event or secondary calibration point), we chose to investigate our data using a molecular clock approach to provide a hypothetical framework for discussion. Prior to estimating divergence times, we used a likelihood-ratio test (Huelsenbeck & Crandall 1997) on Cytb and CO2 to assess rate homogeneity among taxa. This test compares a molecular clock-constrained tree to an unconstrained tree reconstructed in PAUP* 4.0b10 [67] with a null hypothesis of a homogeneous rate of evolution among all branches in the phylogeny. In the present study, the test rejected the null hypothesis for the CO2 data set (LR = 17.11 >> critical value 11.07 with p = 0.05), but not for the Cytb data set (LR = 4.75 << critical value 11.07 with p = 0.05), which indicates that rates of substitution do not vary significantly among branches and that a molecular clock model is appropriate for the Cytb data set.

The ‘‘standard’’ mitochondrial DNA (mtDNA) clock is estimated at 2.3% My^−1^ [68], and has been found empirically to correspond to independent calibrations in many case studies [69]; Therefore, we used this rate in a first approach. This rate, however, is problematic because i) it has been defined for the CO1 gene only and despite being often applied to the evolution of other genes, and ii) it is difficult to apply a rate known in one species to another species because the variance of evolution rates could be important. Moreover, LR tests detect only a few cases where these rates vary. For these reasons, we also use the most extreme rates found in literature for Cytb in insects, i.e. 1.1% [70] and 4.22% [71]. The results will be interpreted using both of these extremes since our main goal is to discriminate between two alternative hypotheses: Is *Caledonula* diversification old (around 37–25 Ma) or relatively recent (< 10Ma)?

To estimate the relative age of divergence of the studied lineages, we used the Bayesian relaxed phylogenetic approach implemented in BEAST 1.4.8 [72] using the best-fitting model as estimated by jModelTest 0.1.1 [58], with the Cytb data set only.

We used a normal distribution for the prior substitution rate, with a mean substitution rate per lineage per million years of 0.0115 (for a substitution rate of 2.3%), of 0.0055 (for a substitution rate of 1.1%) and of 0.0211 (for a substitution rate of 4.22%) and a standard deviation of 0.002. Only the ingroup (genus *Caledonula*) was constrained on the topology and all other relationships were left free to vary so that topological uncertainty was incorporated into posterior estimates of divergence dates.

We confirmed the results by using two independent analyses over 10 million generations, and we sampled every 1000 generations to obtain a maximum of 10,000 samples, as recommended by [72]. The two analyses converged on similar posterior estimates. We then used Tracer 1.4.1 [62] to assess convergence, measure the effective sample size of each parameter, and calculate the mean and 95% highest posterior density (HPD) interval for divergence times. We determined whether a sample size greater than 200 was achieved for all parameters after the analyses. Results of the two runs were combined with LogCombiner 1.4.7 [72], and the consensus tree was compiled with TreeAnnotator 1.4.7 [72].

### 2.6. Geographical distribution, soil diversity and niche modelling

We aimed to test whether certain environmental parameters (soil, climate) could have influenced the distribution of the genus. Ecological niche models (ENM) were constructed using the maximum entropy niche modelling approach implemented in MAXENT [73], [74]. This is one of the best-performing programmes for species distribution models, which it builds based on presence only [75]. Data on absences were therefore not used in the analysis, but we show them to illustrate all the investigated areas (see results section). Environmental data layers were constructed for the 19 BIOCLIM variables (at 30 arc-seconds resolution) in the worldclim dataset. These variables were derived from the interpolation of monthly readings for precipitation and minimum, maximum, and mean temperatures for the period 1950–2000 [76].

## Results

### 3.1. Taxonomy (see Appendix 1)

The genus *Caledonula* is extensively revised here. The type species *C. fuscovittata* is redescribed and three new species are described.

### 3.2. Geographical distribution and soil diversity

**Figure 2:**
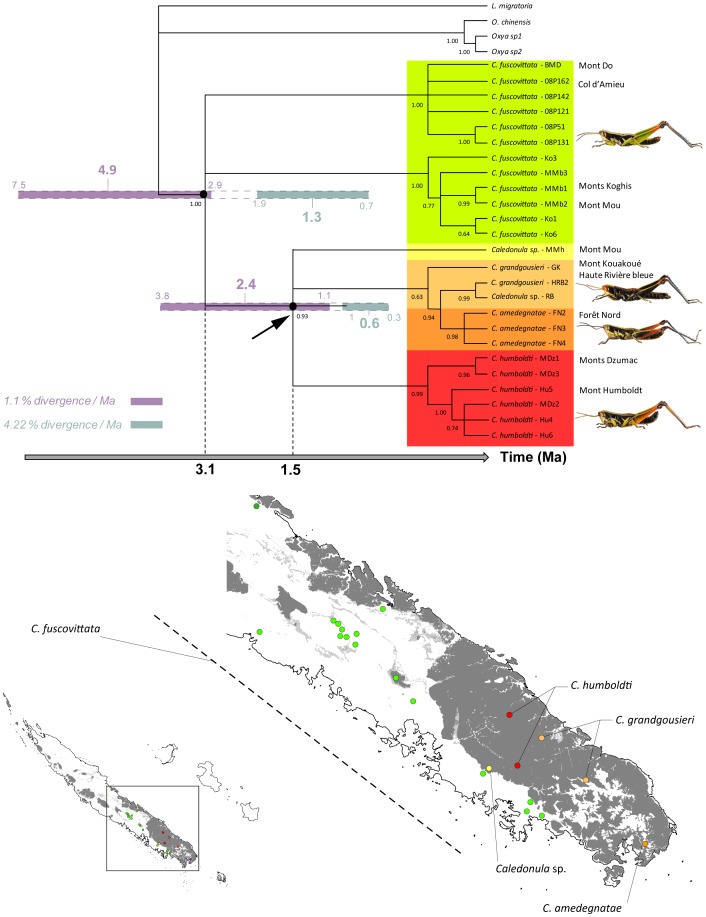
50% majority-rule consensus tree for *Caledonula* obtained from Bayesian analysis of the combined data set (Cytb, CO2, EF1a and H3). The Bayesian posterior probabilities are indicated below branches. The geographical distribution is given at the right of the taxon names. On the topology, pale blue lines represent the dating results obtained with the divergence rate of 4.22%/Ma, and violet lines represent the dating results obtained with the divergence rate of 1.1%/Ma. On the maps, the distribution of ultramafic rocks and corresponding metalliferous soils is indicated in grey, and the species distributions are indicated with the same colour as in the phylogeny.

*Caledonula fuscovittata* has a large distribution with twelve distant localities, always on non-metalliferous soils (Figure 2). Specimens of this species collected on Mont Mou and Mont Koghis (ultramafic massifs) were collected on non-metalliferous soils at the base of these mountains and the species was not found on the ultramafic higher elevations. Mont Do has a heterogeneous soil composition: it is non-metalliferous at the bottom and metalliferous at the top. Except for one specimen showing contradictory information on the labels (GPS data indicates the top of Mont Do, whereas it is labelled as being from the base of Mont Do), all specimens identified as *C. fuscovittata* are from non-ultramafic soils only.

All other species were located on ultramafic soils in the South, and each is known only from one or two close localities (Figure 2), suggesting that they are highly microendemic.

### 3.3. Phylogenetic analysis

Our data matrix consisted of 101 DNA sequences (Cytb: 27, CO2: 27, EF1a: 24, H3: 23) from 29 terminals after discarding data for CO1 (due to suspicion of pseudogene amplification) and for 28S and ITS1 (uninformative) (Table S1). All remaining coding sequences could be translated into amino acids with no evidence of pseudogenes.

The Bayesian and parsimony analyses resulted in very similar relationships amongst taxa (Figure S1 and S2), with slight differences that did not affect the results of the dating events.

*Caledonula fuscovittata* appears paraphyletic in parsimony analysis and polyphyletic (unresolved basal polytomy) with Bayesian analysis. The other species found on ultramafic soils are grouped in a well-supported clade (bootstrap  = 72%, posterior probabilities  = 0.86). *Caledonula grandgousieri* and *C. amedegnatae*, are either the sister group to *C. humboldti* (parsimony) or form a polytomy with it (Bayesian). The other species are clearly monophyletic according to the phylogenetic analyses. The analyses also suggest that an additional new species may exist on the high summit of Mont Mou, represented by only a juvenile in our sampling; this species is sister to the other species of the clade found in ultramafic soils.

Preliminary separate analyses show that the observed pattern from the total data set is not biased by the presence of pseudogenes or other artefacts. The non-monophyly of *C. fuscovittata* is confirmed by all the analyses, which split the species into two well-supported clades: one groups specimens from Col d’Amieu and from the base of Mont Do, and the other clade groups specimens from the base of Mont Mou and Monts Koghis. The clade including all other species was also recovered in separate analyses (Figure 2).

### 3.4. Molecular dating

The two combined beast runs yielded high effective sample sizes (> 200) for all relevant parameters, indicating adequate sampling of the posterior distribution.

Using a divergence rate of 2.3%, the divergence of *Caledonula* took place around 2.4 Ma (95% confidence interval: 1.4–3.6 Ma), and the divergence of species found on metalliferous soils around 1.1 Ma (95% confidence interval: 0.5–1.8 Ma).

The tentative use of a greater divergence rate (4.22%) led to more recent ages: 1.3 Ma (0.7–1.9 Ma) for diversification of the genus and 0.6 Ma (95% confidence interval: 0.3–1 Ma) for species on metalliferous soils (interval in pale blue on Figure 2). At the other extreme use of a lower divergence rate (1.1%) led to more ancient ages: 4.9 Ma (2.9–7.5 Ma) for diversification of the genus and 2.3 Ma (95% confidence interval: 1.1–3.9 Ma) for diversification of the group located on metalliferous soils (interval in violet on Figure 2).

Taking into account these extreme rates, diversification of the genus *Caledonula* is presumed to have occurred around 3.1 Ma (4.9–1.3 Ma) and that of the clade on metalliferous soil around 1.5 Ma (2.4–0.6 Ma) (Figure 2). This relatively recent diversification could explain the non-monophyly of *C. fuscovittata*. There are, however, no morphological differences between the specimens from both clades, which could indicate putative cryptic species or very recent species divergence. However, even if we consider two species from the *C. fuscovittata* group, the diversification and the degree of microendemism is still greater on metalliferous soils.

### 3.5. Ecological niche models

**Figure 3:**
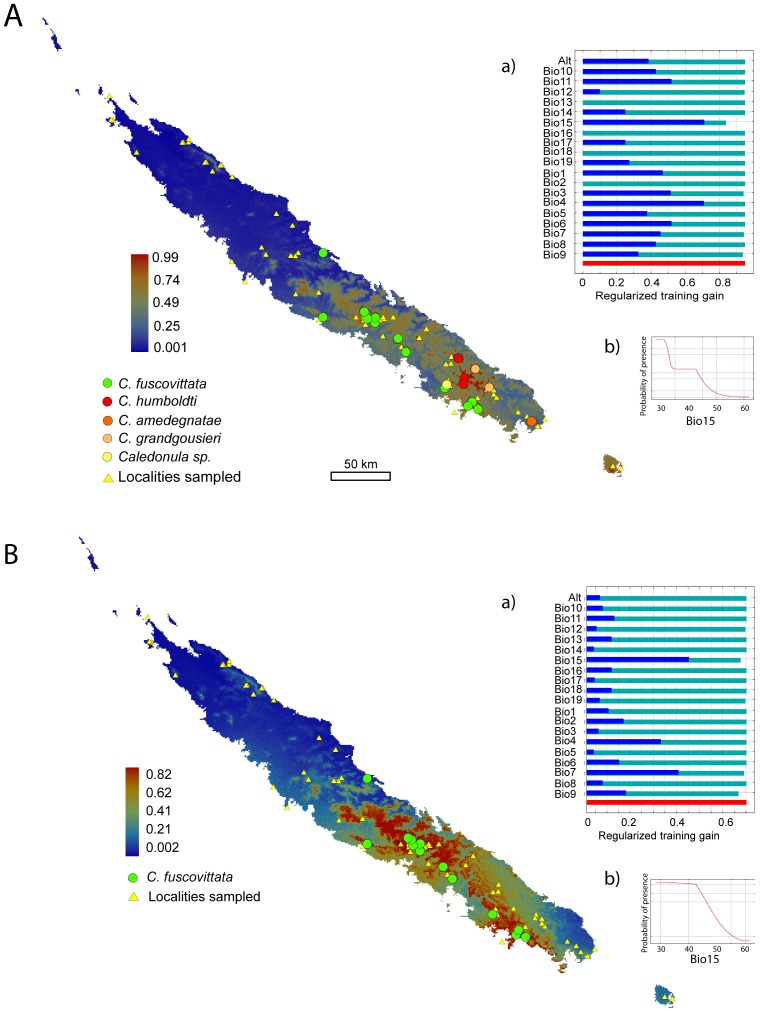
Predicted distribution of the genus *Caledonula* (A) and of the species *Caledonula fuscovittata* (B) constructed from presence data using MAXENT. Results are presented for logistic probabilities of occurrences ranging continuously from low to high. Warmer colours show areas with predicted better conditions. Dots show species’ sampling locations; triangles indicate all other sampled sites in New Caledonia, to indicate those where *Caledonula* was not found. For each map, a) shows the Regularized Training Gain under 100 Jackknife turns (green: without variable; blue: with only one variable; and red: with all variables); b) shows the response curves of BIO 15, the variable that contributes most to these models.

The Ecological Niche Models (ENM) for the genus *Caledonula* and for the species *C. fuscovittata* are shown in Figure 3. In both cases MAXENT appeared to perform well. The ENM for all the species of the genus was based on 26 presence records and had an AUC (Area under Receiver Operating) of 0.884. The ENM of *C. fuscovittata* was based on 19 presence records with an AUC of 0.885 (See [73]).Both ENMs indicated areas with suitable environmental conditions from East to West coast, on both soil types, from sea level to mountaintops. But these suitable areas were constrained from the centre to the south, including Ile des Pins, showing clearly that climate could be an important factor to explain why *Caledonula* distribution is limited to the central-southern part of the island. As can be seen in Figure 3, the absence of *Caledonula* in the North is not a sampling artefact. Indeed, several sites in potentially suitable habitats were sampled, without *Caledonula* ever being found. Different kinds of soils occur in different climatic conditions; therefore, there is no confounding effect of soil on climatic niche description or vice versa.

In both cases, the variable that contributed most to the MAXENT model was BIO 15 (precipitation seasonality) with 51% for the genus and 63% for *C. fuscovittata*. The response curve showed that the best predicted environmental conditions were in areas where precipitation is less seasonal. The response to BIO 9 by *C. fuscovittata* follows the same pattern, i.e. the probability of presence is higher in areas where the mean temperature of the driest quarter is lowest. A jackknife test confirms the importance of this variable. For the genus *Caledonula*, BIO 15 is the variable with highest gain when used in isolation and the one that decreases the most the gain when it is omitted. For *C. fuscovittata* alone, BIO 15 is also the variable with the highest gain when used in isolation, but the variable that most decreases the gain when omitted is BIO 9 (mean temperature of the driest quarter). All this indicates that species of *Caledonula* are probably limited by extreme dryness in strongly seasonal climates.

## Discussion

New Caledonia has recently undergone a change in biogeographical status, from Gondwanan refuge to old Darwinian island [8]. The question of the origin of a diversification in New Caledonia is thus crucial to determining whether a group is as old or even older than the island. The age of origin of the *Caledonula* grasshoppers in New Caledonia has been conservatively estimated by using extreme rates of molecular evolution, either very slow or very fast. In either case, the results support a relatively recent diversification of not much more than six million years ago, and likely around three million years ago (Figure 2). This inference fits the new paradigm of New Caledonia biogeography, with recolonisation of the newly emerged main island later than 37 My ago, either by long or short distance dispersal [8], [9]. Although this dating was obtained without calibration points, in accordance with [69] or [77], the extreme rates employed here (very slow or very fast) encompass those obtained with datings performed with relaxed molecular clocks and more sophisticated calibrations (e.g., [9], [23]). Pushing the present dating back to the land emersion (37 My) or to the separation of the geological basement from Australia (80 My) would imply molecular rates never met before, ten times or more slower than any rate ever carefully estimated with fossils or paleogeography for any organism. The molecular differences among *Caledonula* species and with the outgroups cannot therefore be consistent with a scenario of an old origin of the genus on the island.

A relatively recent age is also significant regarding the environmental factors that are generally considered to explain the intensity of speciation in New Caledonia. A recent age is at odds with the scenario usually put forward to explain adaptive radiation on metalliferous soils by an early arrival of organisms on an island still mainly covered by ophiolithic rocks, before they were widely eroded by subtropical wet climates [28], [78]. In this scenario, an early arrival, not long after the island emersion 37 My ago, would imply an “all or nothing” evolutionary scenario like “adapt or perish”, on an island where all soils were metalliferous. It can explain why some old local groups were ancestrally adapted or preadapted to metalliferous soils [3], [26], [79]. In the present case, the grasshoppers diversified more than 20 million years after emersion, when most of the land was already freed from the cover of ophiolithic rocks for millions of years [10], [28]. Therefore, it relaxes the assumption that there was a historical constraint for adaptation to metalliferous soil for *Caledonula*, which could be recent and possibly secondary after colonization of the island, depending on the history of the genus.

Though often expected, diversification in relation to metalliferous soils is still poorly documented for New Caledonian organisms, with the exception of plants [79]. In the case of *Caledonula*, this relationship was completely unknown until now, with only a few old taxonomic mentions of this genus in the literature, and the only species described in the early twentieth century was the most common species, *C. fuscovittata*, found only on non metalliferous soils [29], [30], [80], [81].The phylogenetic tree strongly suggests that most of the diversification of *Caledonula* took place on metalliferous soils. Four species are known to occur on metalliferous soils, compared to one occurring on volcano-sedimentary soils. Speciation seems to have occurred in allopatry, since sister-groups are distributed on different areas close to each other. The topology is not conclusive enough to distinguish between different scenarios of origin because the clades on each kind of soil, metalliferous or not, are branching on a trichotomy. Either the clade was ancestrally able to deal with the ecological constraints of metalliferous soils, or it developed first on non-metalliferous soils and shifted to metalliferous soils later. In either case, the most strictly microendemic species are located on metalliferous soils and they constitute a monophyletic clade. Morphological evidence also suggests specialization and adaptation for these species, which are more robust and have very large mandibles, related to feeding behaviour on specifically hard-leaved Poaceae, compared to the other species of the genus and related acridids. Such a relationship with soils was expected in grasshoppers because they are phytophagous insects that directly depend on plants, themselves strongly constrained by stressed soils [79], [82], [83]). In other insects, a relationship to metalliferous soils has been found in caddisflies with aquatic larvae that diversified first and more on such soils [26]. Conversely, such a relationship was not found in other non-aquatic saprophagous insects [7], [21]. In *Agnotecous* crickets, the relationship is of similar complexity to that of *Caledonula*, since the species of the former genus are found on every kind of soil, but show different distributions and evolutionary characteristics in each case [23].

Therefore, the diversification of *Caledonula* appears to be at least partly related to soil type. However, given the peculiar distribution of *Caledonula*, other causes or constraints on diversification have to be considered. The climate niche model shows that the genus is constrained to high rainfall areas with low seasonality. The results remain similar when considering only the most abundant and widely distributed species, *C. fuscovittata,* which is not found on metalliferous soils. More generally than for soils, we can conclude that the whole genus is restricted to areas where the climate is wet but not very seasonal. This is an interesting and unexpected result since many distributions in New Caledonia are limited to northern versus southern half of the elongated main island (e.g., [20]), a situation generally interpreted in terms of unknown historical constraints (P. Bouchet, pers. comm.) or in terms of relationships with the very large southern area of metalliferous soils. Here we show that such distributions could also be correlated with a climatic parameter that is never mapped in atlases or books [84], since it depends on the combination of different variables, and whose role was therefore not suspected a priori. Bioclimatic niche modelling studies have been conducted several times in New Caledonia and showed that climate plays a role in shaping distributions, but none of these studies showed such a strong constraint explaining a clear-cut distributional pattern [85], [86].

Concerning the hierarchy or the temporal succession of the roles for soils versus climate in *Caledonula* diversification, we can only remark that climate probably had an earlier influence because it affects all the species including *C. fuscovittata*. The genus would have diversified later, including with the presumptive adaptation to metalliferous soils. The reasons why adaptation to metalliferous soils has led to increased speciation in the genus (four out of five species) remains unclear and will require further studies. One explanation could involve the complexity of the landscape covered by ophiolithic rocks giving birth to metalliferous soils. Ophiolithic rocks actually erode faster than other types, generating a fast-evolving and dissected landscape [28], which could in turn generate more possibilities of allopatric speciation, by increasing the opportunity of population fragmentation according to orography and climate changes. This hypothesis was already proposed in the case of the cricket genus *Agnotecous* [23], where the recent and more narrowly distributed species mostly occurred on metalliferous soils. Another explanation could call for the occurrence of adaptive speciation, considering the visible modifications of head and mandible morphology in species living on metalliferous soils. These hypotheses are not, however, mutually exclusive because speciation could be favoured both by population fragmentation in complex landscapes and adaptation to different food plants.

In conclusion, microendemism in *Caledonula* grasshoppers is clearly related to both climatic and to soil constraints. The distributions appear to be restricted by ecological and evolutionary constraints. Distributions have been, and still are limited by seasonality, but they also resulted from the fragmentation of probably larger ancestral distribution areas after allopatric speciation. In this respect, *Caledonula* grasshoppers are an interesting and original model for the study of microendemism in New Caledonia, quite different from those previously studied, in which niche conservatism dominates or in which metalliferous soils are an ancient resource, rather than an opportunity for recent adaptation.

## Appendix 1: Taxonomy of *Caledonula*

Family Acrididae

Subfamily Cyrtacanthacridinae Kirby, 1910.

Genus *Caledonula* Uvarov, 1939.

*Caledonula* Uvarov, 1939: 459.

   Synonym. *Caledonia* Willemse, 1923: 103. Name preoc cupied, renamed *Caledonula* (Uvarov 1939).

Type species. Caledonia fuscovittata Willemse, 1923.

Redescription

Body bicoloured, yellowish-brown dorsally and ventrally, sides with a wide black longitudinal band. Face and mouthparts yellowish. Dorsal part of tegmen yellow, lateral part black, continuing the black band along the whole body. Leg colouration variable between species. Body quite robust. Foveola absent; antennae filiform, as long as head and pronotum. Anterior margin of pronotum almost straight; posterior margin thickened, with a median incision; median carina weak; with 2–3 distinct sulci; lateral carinae weak, slightly divergent posteriorly; dorsal cuticle with rough patches on anterior and posterior edges, the posterior rough area longer than the anterior one. Tegmina short in both sexes, apex rounded, very sclerotised, with faint longitudinal veins. Prosternum armed with a trilobate tubercle process. Dorsal carina of hind femora prolonged by a small apical spine; lateral lobes of knees ending in a small spine. Hind tibiae with bicoloured spines, their base lighter than apex. First segment of hind tarsomeres twice as long assecond one.

### Caledonula fuscovittata (Willemse, 1923) (Figures 4, 5, 6, 7)

Caledonia fuscovittata Willemse, 1923: 103.

*Caledonula fuscovittata –* Uvarov, 1939.

**Figure 4:**
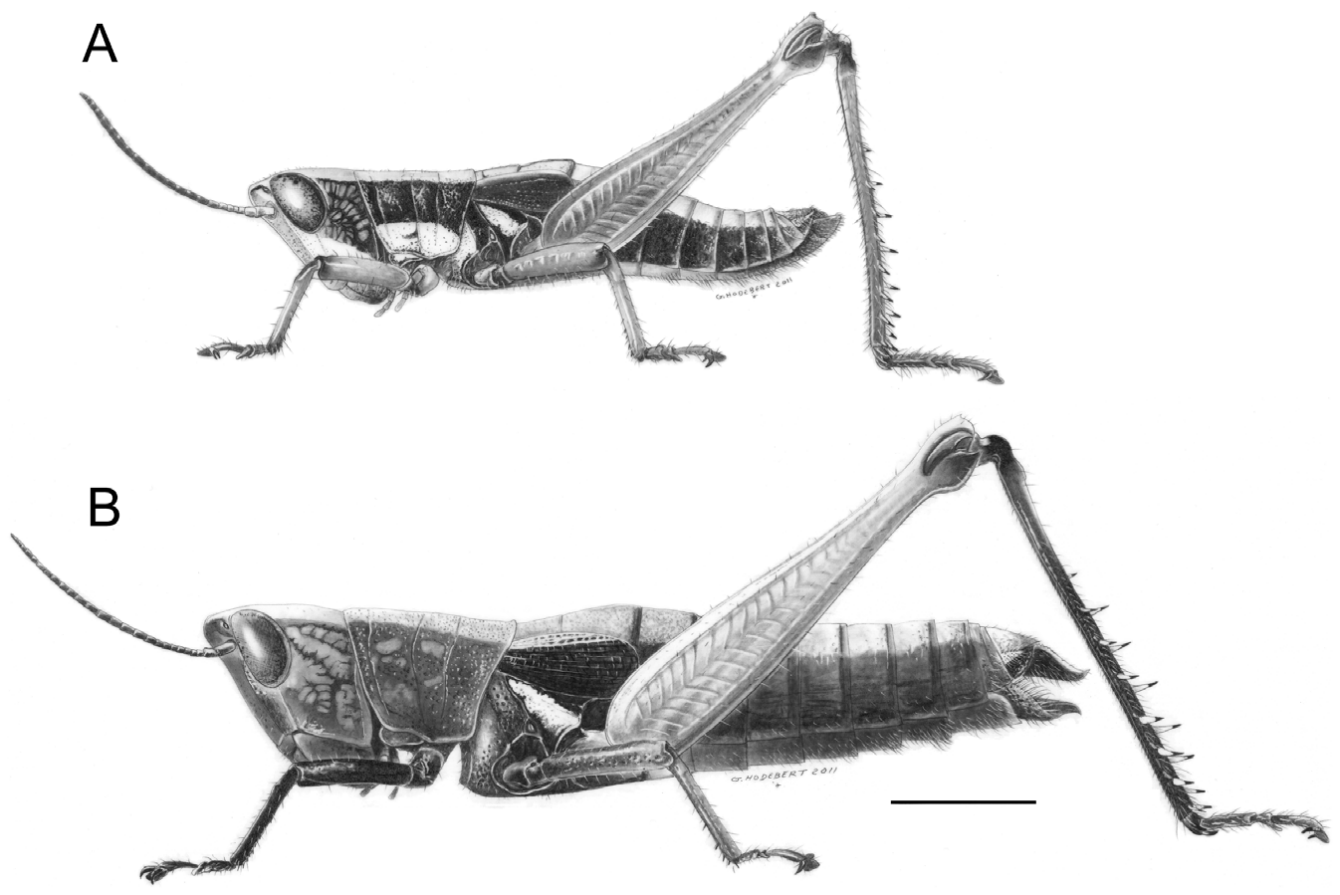
*Caledonula fuscovittata*, male (A) and female (B) habitus. Scale bar: 5 mm. Drawings by Gilbert Hodebert (MNHN).

**Figure 5:**
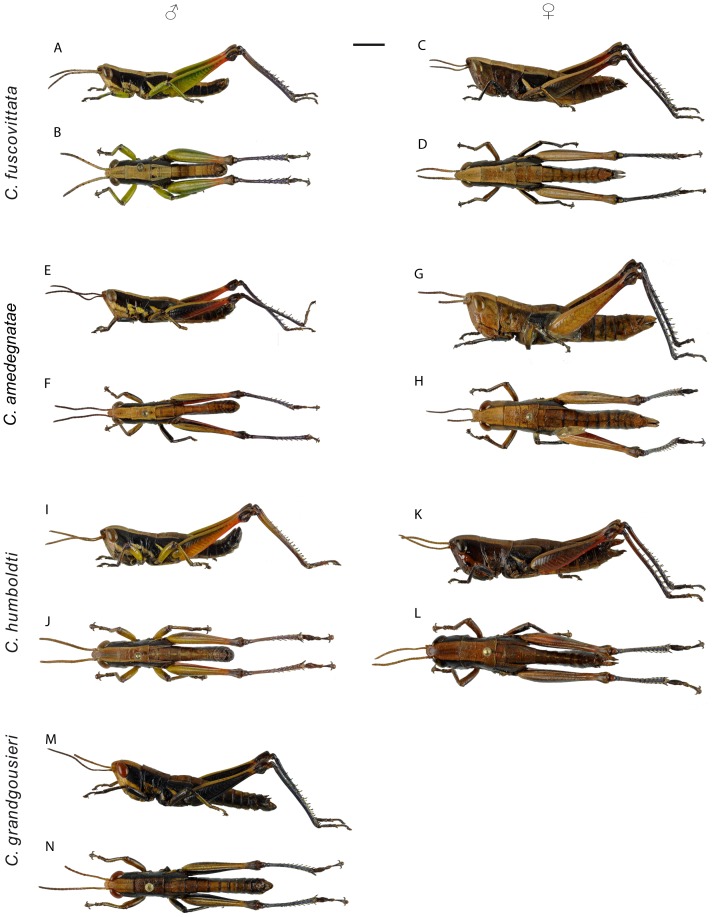
*Caledonula* species in dorsal and lateral view. *C. fuscovittata* (A-D), *C. amedegnatae* n. sp. (E-H), *C. humboldti* n. sp. (I-L) and *C. grandgousieri* n. sp. (M-N). Left: ♂, right: ♀. Scale bar: 5 mm.

*Differential diagnosis.* Size small for the genus, slender, differing from other species by vivid green leg colouration in males (Figure 5).

**Figure 6:**
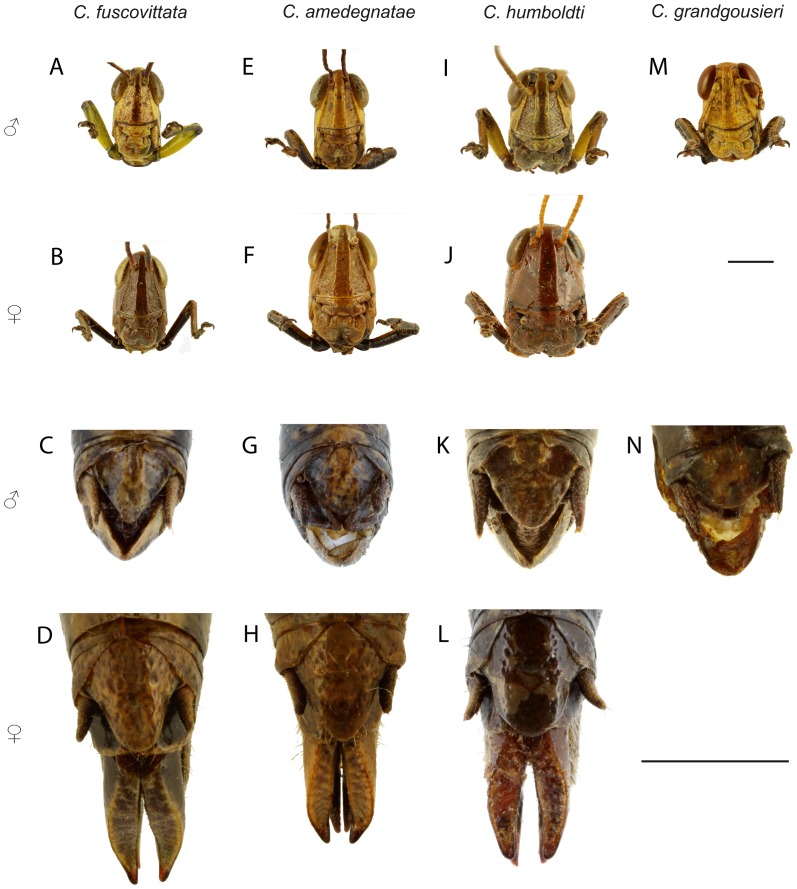
*Caledonula* species: face (upper part) and epiproct in dorsal view (lower part). *C. fuscovittata*: (♂: A, C; ♀: B, D), *C. amedegnatae* n. sp. (♂: E, G; ♀: F, H), *C. humboldti* n. sp. (♂: I, K; ♀: J, L) and *C. grandgousieri* n. sp. (♂: M, N). Scale bars: 2 mm.

*Redescription.* Size small, head narrow for the genus, with weak mandibles. General colour pattern close to other *Caledonula* species, but differing in leg colouration (Figures 5, 6).

**Figure 7:**
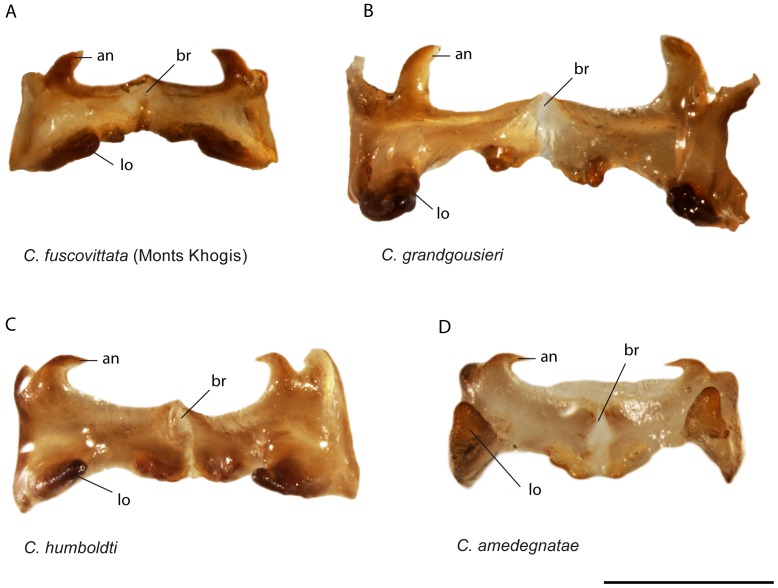
*Caledonula* species, male genitalia in dorsal view. (an: ancorae; br: bridge; lo: lophi). Scale bars: 1 mm.

Male. Fore and median legs vivid green, knees black. Hind femora mostly vivid green, distal part red and knees black; hind tibiae purple, their bases dark purple and lighter toward apex (Figure 5A, B). Green parts in some old specimens including the HT have turned dark brown or black. Dorsal side of hind tibiae with 7-8 spines (m = 7.3; n = 38) on outer edge and 7-9 spines (m = 8; n = 39) on inner edge. Epiproct triangular, apex acute with an elongated impression (Figure 6C). Male genitalia (Figure 7A): Epiphallic lophi convergent, flattened, with basal membrane continuous toward the bridge; ancorae convergent and very acute, narrowed apically.

Female. Body colour almost uniformly brownish, with a dorsal light brown band and darker lateral brown bands, except a yellowish line on metathorax epimeron. Tegmina black with a dorsal light brown band. Distal part of hind femora red, dorsal part cream, knees dark brown (Figure 5C, D). Hind tibiae dorsal side with 7-8 spines (m = 7.3, n = 31) on outer edge and 7-8 spines (m = 7.8, n = 31) on inner edge. Valves of ovipositor long and parallel, except for the curved apex (Figure 6D). Basis of epiproct narrow with an elongated impression, apex rounded (Figure 6D).

*Measurements (in mm).* 20♂, 21♀. Body length: 18.5–21.2 (m = 19.9) (♂), 24–31.7 (m = 27.7) (♀); femur III length: 10.1–13.6 (m = 12.3) (♂), 13.5–18.2 (m = 15.5) (♀); tegmina length: 2.7–4.2 (m = 3.5) (♂), 3.7–5 (m = 4.5) (♀); pronotum length: 3.5–4.2 (m = 3.9) (♂), 4.8–6.5 (m = 5.4) (♀).

*Type material*. Lectotype. ♂ dissected (*Montague*) (BMNH) (examined). Paralectotype. ♀ (*Montague*) (BMNH). Original type series consisted of 2♂ and 1♀ syntypes from Grande Terre, Canala (Willemse, 1923).

*Other material examined*. NEW CALEDONIA: 3♂, Grande Terre, Réserve naturelle du Mont Mou (22°04'28.5''S; 166°19'50.7''E), 360 m, 26.iv.2009 (*R. Nattier*) (MNHN-CAELIF900-902), 1♂, 2♀ same information (MNHN); 1♀, Grande Terre, Réserve naturelle du Mont Mou (22°04'30''S; 165°19'52''E), 390 m, 22.v.2008 (*T. Robillard* &* F. Muller*) (MNHN); 2♂, Grande Terre, Monts Koghi (22°10'44''S; 166°30'31''E), 500 m, 06.v.2008 (*T. Robillard* & *F. Muller*) (MNHN-CAELIF903-904), 13♂, 7♀, 3 juveniles same information (MNHN); 1♂, Grande Terre, Monts Koghi (22°10’39’’S; 166°30’29.2’’E), 480 m, 22.iv.2009 (*R. Nattier*) (MNHN-CAELIF905), 6♂, 3♀ same information (MNHN); 1♂; Grande Terre, Monts Koghi, 500 m, 11.ii.1994 (*L. Desutter-Grandcolas*) (MNHN); 2♀, Grande Terre, Monts Koghi, 05.iii.1968 (*J. Chazeau*) (IRD NOUMEA); 3♂, 2♀, Grande Terre, Monts Koghi, 490 m, 09.viii.1979 (*Nishida*) (BPBM); 1♂, Grande Terre, Col d’Amieu (21°35'17''S; 165°47'56''E), 460 m, 11.v.2008 (*T. Robillard* & *F. Muller*) (MNHN-CAELIF906), 5♂, 3♀, 1 juvenile, same information (MNHN); 1♂, Grande Terre, Col d’Amieu (21°34'29''S; 165°47'24''E), 370 m, 09.v.2008 (*T. Robillard* &* F. Muller*) (MNHN-CAELIF907), 1♀, 1 juvenile, same information (MNHN); 1♂, Grande Terre, Col d’Amieu (21°36'52''S; 165°47'45''E), 430 m, 11.v.2008 (*T. Robillard* & *F. Muller*) (MNHN-CAELIF908); 1♂, Grande Terre, Col d’Amieu (21°35'26''S; 165°47'45''E), 420 m, 11.v.2008 (*T. Robillard* & *F. Muller*) (MNHN-CAELIF909), 2 juveniles, same information (MNHN); 1♂, Grande Terre, Col d’Amieu (21°33'28''S; 165°46'01''E), 690 m, 11.v.2008 (*T. Robillard* & *F. Muller*) (MNHN-CAELIF910), 1♀, same information (MNHN); 1♀, Grande Terre, Col d’Amieu, 450–550 m, 18.ii.1994 (*L. Desutter-Grandcolas*) (MNHN); 1♂, 1 juvenile, Grande Terre, Col d’Amieu (21°34'24''S; 165°47'03''E), 450 m, 13.v.2008 (*T. Robillard* &* F. Muller*) (MNHN); 5 juveniles, Grande Terre, Col d’Amieu (21°33'52''S; 166°46'07''E), 515 m, 08.v.2008 (*T. Robillard* &* F. Muller*) (MNHN); 1♂, 1 juvenile, Grande Terre, Col d’Amieu (21°34’40.8’’S; 165°47’36.9’’E), 690 m, 28.iv.2009 (*R. Nattier*) (MNHN); 2♂, 2♀, Grande Terre, Col d’Amieu (21°35’47.2’’S; 165°46’37.0’’E), 680 m, 01.v.2009 (*R. Nattier*) (MNHN); 2♂, 1♀, Grande Terre, Col d’Amieu (21°35’’12.5’S; 165°46’25.7’’E), 470 m, 01.v.2009 (*R. Nattier*) (MNHN); 2♂, Grande Terre, Col d’Amieu (21°35’08.0’’S; 165°47’27.6’’E), 480 m, 27.iv.2009 (*R. Nattier*) (MNHN); 1♀, Grande Terre, Col d’Amieu (21°33’55.8’’S; 165°45’35.2’’E), 460 m, 29.iv.2009 (*R. Nattier*) (MNHN); 1♀, Grande Terre, Col d’Amieu (21°34’10.8’’S; 165°45’42.0’’E), 440 m, 29.iv.2009 (*Nattier*) (MNHN); 1 juvenile, Grande Terre, Col d’Amieu (21°36’01.9’’S; 165°46’29.3’’E), 680 m, 01.v.2009 (*R. Nattier*) (MNHN); 1♀, Grande Terre, Col d’Amieu (21°34’22.8’’S; 165°46’35.2’’E), 30 m, 02.v.2009 (*R. Nattier*) (MNHN); 2♂, Grande Terre, Col d’Amieu, 12-13.iii.1986 (*J. Boudinot*) (MNHN); 1♂, ♂, Grande Terre, Col d’Amieu, 650 m, 21.iii.1986 (*Gressitt & Maa*) (BPBM); 2♂, Grande Terre, Col d’Amieu (21°37S; 165°49E), 08.ii.1998 (*J. Chiffaud* & *J. Mestre*) (MNHN-CAELIF***); 2♀, Grande Terre, Col d’Amieu (21°37S; 165°49E), 01-15.iii.1998 (*J. Chiffaud* & *J. Mestre*) (MNHN); 1♂, Grande Terre, Réserve naturelle du Mont Do (21°45'28.71''S; 166°00'00.51''E), 933 m, 08.xi.2007 (*S. Cazeres*) (MNHN-CAELIF911); 1♂, 3♀,2 juveniles, Grande Terre, Mont Rembaï (9.2 km North-East of Col d’Amieu on road 5), 375–675 m, 23.ix.1979 (*Nishida*) (BPBM); 1♀, Grande Terre, Yahoué, 22.ii.1962 (*Krauss*) (BPBM); 1♀, Grande Terre, Yahoué, 22.i.1963 (*Yoshimoto*) (BPBM); 1♂, Grande Terre, Yahoué, ii.1978 (*Krauss*) (BPBM); 1♂, 1♀, Grande Terre, Saint Louis Valley, 17-22.iii.1945 (*Milliron*) (BPBM); 1♂, Grande Terre, Saint Louis Valley, iv.1939 (*Jaubert*) (MNHN); 1♂, 1♀, Grande Terre, near Bouloupari, 25.ii.1945 (*Milliron*) (BPBM); 1♀, Grande Terre, Sarraméa, 12.ii.1963 (*Yoshimoto*) (BPBM); 1♀, Grande Terre, Bourail, 1902 (*Méray*) (MNHN).

### *Caledonula grandgousieri* Nattier sp. nov. urn:lsid:zoobank.org:act:26A5EF4F-AC4D-4B83-9BB4-C45FEB477C5B (Figures 5, 6, 7)

*Differential diagnosis (male).* Size large, mandibles strong, similar to *Caledonula humboldti*, but differing in the form of male genitalia and by having a more uniform and darker colouration (Figure 5).

*Description.* Size large for the genus, head wide with strong mandibles. General colour pattern close to other species, but darker and more uniform (Figure 5, 6).

Male. Fore femora and tibiae black on outer side and yellowish on inner side; median femora and tibiae yellowish with thin black bands on outer side and black on inner side; hind femora black on inner and outer side except proximal and distal parts; knees black; hind tibiae dark purple (Figure 5K, L). Hind tibiae dorsal side with 9 (n = 4) spines on outer edge and 8-9 (m = 8.5; n = 4) spines on inner edge. Epiproct triangular, apex rounded with an oval/rounded impression (Figure 6N). Male genitalia (Figure 7B): epiphallic bridge little sclerotised; lophi slightly pointed and not convergent, ancorae straight, with acute and curved apex.

Female. Unknown.

*Measurements (in mm).* 2**♂**. Body length: 25.5–25.8 (m = 25.7); femur III length: 14.4–15 (m = 14.7); tegmina length: 4.5–5.1 (m = 4.8); pronotum length: 4.4–4.6 (m = 4.5).

*Material examined. Holotype,* ♂, NEW CALEDONIA: Grande Terre, Parc de la Rivière Bleue, Haute Rivière Bleue, v.iii.1986 (*J. Boudinot*) (MNHN-CAELIF912). *Other material studied,* NEW CALEDONIA: 1♂, Grande Terre, Mont Kouakoué (21°57'25,69''S, 166°32'17,40''E), 1280 m, 17.iii.2009 (*G. Kergoat*) (MNHN-CAELIF913).

*Etymology.* The species name refers to the large size and the wide head of the species.

*Distribution.* New Caledonia, Grande Terre, Province Sud: Parc de la Rivière Bleue and Mount Kouakoué.

***Caledonula humboldti*** **Nattier sp. nov. urn:lsid:zoobank.org:act:5C761942-81F6-4248-99E7-C751C9D9ACBB** (Figures 5, 6, 7)

*Differential diagnosis (male).* Close to *C. grandgousieri* by large size and stocky shape, but differing by the yellow colouration of fore and median femora, and by the more contrasted colours of hind legs (Figure 5).

*Description.* Size large, head wide for the genus with strong mandibles. General colour pattern close to that of other species, differing by leg colouration (Figures 5, 6).

Male. Fore femora yellow; tibiae yellow, their external side dark brown; knees black. Median legs yellow, knees black; hind femora orange-red ventrally, their dorsal edge yellowish, with a lateral black band narrowing toward black knees (Figure 5I, J). Hind tibiae brown, their dorsal side with 7-10 spines (m = 8.4; n = 14) on outer edge and 8-10 spines (m = 8.9; n = 14) on inner edge. Epiproct triangular, apex rounded with an oval impression (Figure 6K). Male genitalia (Figure 7C): epiphallic bridge weakly sclerotised; lophi convergent, conical and wide basally; ancorae curved, convergent and pointed.

Female. Body almost uniformly brownish, light brown dorsally, darker laterally, with a yellow line on the metathorax epimeron. Forewings light brown, except a lighter dorsal brown band (Figure 5K, L). Hind tibiae dorsal side with 7-9 spines (m = 8.2; n = 10) on outer edge and 9 spines (n = 10) on inner edge. Valves of ovipositor long, slightly divergent toward the end, apex curved (Figure 6L). Basis of epiproct wide with an oval impression, apex rounded (Figure 6L).

*Measurements (in mm).* 7♂, 5♀. Body length: 22.2–24 (m = 22.9) (♂), 29.8–32.1 (m = 31) (♀); femur III length: 13.2–14.8 (m = 13.7) (♂), 17–18 (m = 17.5) (♀); tegmina length: 4.1–5.1 (m = 4.5) (♂), 5–6.5 (m = 5.7) (♀); pronotum length: 4.3–4.7 (m = 4.5) (♂), 5.5–6 (m = 5.8) (♀).

*Material examined. Holotype,* ♂, NEW CALEDONIA: Grande Terre, Mont Humboldt (21°52'57,4''S, 166°24'45,3''E), 13.x.2009 *(P. Grandcolas)* (MNHN-CAELIF914). *Allotype,* ♀, NEW CALEDONIA: Grande Terre, same locality, date and collector as holotype (MNHN-CAELIF926). *Paratypes,* NEW CALEDONIA: 3♂, 1♀, Grande Terre, Mont Humboldt (21°52'57,4''S, 166°24'45,3''E), 1343m, 13.x.2009 *(P. Grandcolas)* (MNHN-CAELIF915-918); 1 ♀, Grande Terre, Mont Humboldt (21°52'48,6''S, 166°25'14,0''E), 1564 m, 14.x.2009 (*P. Grandcolas*) (MNHN-CAELIF919); 2♂, Grande Terre, Mont Humboldt (21°52'50,4''S, 166°25'08,5''E), 1510 m, 14.x.2009 (*P. Grandcolas*) (MNHN-CAELIF920-921); 2♂, 1♀, Grande Terre, Mont Humboldt, 1350 m, 10-11.ii.2005 (*S. Cazeres* & *C. Mille)* (IAC). *Other material studied,* NEW CALEDONIA: 1♀, Grande Terre, Mont Humboldt, Païta (21°52’58.01’’S, 166°24’45.01’’E), 1350 m, 15.ii.2006 (*S. Cazeres*) (IAC); 2 juveniles, Grande Terre, Mont Humboldt (21°52'48,6''S, 166°25'14,0''E), 1564 m, 14.x.2009 (*P. Grandcolas*) (MNHN-CAELIF922-923); 2 juveniles, Grande Terre, Monts Dzumac (22°01'09,9''S, 166°27'52,5''E), 878 m, 12.v.2009 (*R. Nattier)* (MNHN-CAELIF924-925); 1 juvenile, Grande Terre, Monts Dzumac (22°03'18,9''S, 166°26'55,7''E), 474 m, 12.v.2009 (*R. Nattier)* (MNHN-CAELIF934); 1 juvenile, Grande Terre, Mont Humboldt, 1350 m, 10-11.ii.2005 (*S. Cazeres* & *C. Mille)* (IAC).

*Etymology.* The species name refers to the type locality.

*Distribution.* New Caledonia, Grande Terre, Province Sud: Mount Humboldt and Mount Dzumac.

***Caledonula amedegnatae*** **Nattier sp. nov. urn:lsid:zoobank.org:act:55EAE7A4-6002-4BD6-821F-AFA01AAE77D5** (Figures 5, 6, 7)

*Differential diagnosis (male).* Similar to *C. fuscovittata* in terms of small size and shape, but differing in colouration by being more uniform and darker, except for the distal part of hind femora, which is red in both species (Figure 5).

*Description.* Size small, head narrow for the genus, with weak mandibles. General colour pattern close to other species, differing by leg colouration (Figures 5, 6).

Male. Fore legs black on external side, brown on internal side. Median legs mostly yellow on external side and black on internal side. Hind femora black, their dorsal edge yellowish, ventral edge and distal region red, knees black; hind tibiae dark purple (Figure 5E, F). Hind tibiae dorsal side with 8-9 spines (m = 8.7; n = 6) on outer edge and 7-9 spines (m = 8.3; n = 6) on inner edge. Epiproct triangular, apex rounded with an oval impression (Figure 6G). Male genitalia (Figure 7D): epiphallic bridge weakly sclerotised; lophi not convergent; ancorae thin and curved, convergent and pointed.

Female. Body colour almost uniformly brownish, with a dorsal light brown band and darker lateral bands except for a yellowish line on the metathorax epimeron. Internal part of fore and median tibiae brown, external part dark brown. External part of hind femora brown, external part red, darker towards the black knees; hind tibiae black. Forewings black with a dorsal light brown band (Figure 5G, H). Hind tibiae dorsal side with 9 spines (n = 2) on outer edge and 10 spines (n = 2) on inner edge. Valves of ovipositor long and parallel, their apex curved (Figure 6H). Basis of epiproct narrow with an oval impression, apex rounded (Figure 6H).

*Measurements (in mm).* 3♂, 1♀. Body length: 21.1–23.4 (m = 22.2) (♂), 23.2 (♀); femur III length: 12.7–13.2 (m = 12.9) (♂), 15.6 (♀); tegmina length: 3.7–4.2 (m = 3.8) (♂), 5.1 (♀); pronotum length: 3.8–3.9 (m = 3.87) (♂), 4.8 (♀).

*Material examined.* Holotype, ♂, NEW CALEDONIA: Grande Terre, Réserve de la Forêt Nord, Yaté (22°19’03.5’ S, 166°54’58.7’’E), 335 m, 08.v.2009 (*R. Nattier)* (MNHN-CAELIF927). *Allotype,* ♀, NEW CALEDONIA: Grande Terre, same locality, date and collector as holotype. *Paratypes,* NEW CALEDONIA: 1♂, Grande Terre, Réserve de la Forêt Nord, Yaté (22°18’58.7’’ S, 166°55’13.6’’E), 390 m, 08.v.2009 *(R. Nattier*) (MNHN-CAELIF928). *Other material studied,* NEW CALEDONIA: 3 juveniles, Grande Terre, Réserve de la Forêt Nord, Yaté (22°19’03.5’ S, 166°54’58.7’’E), 335 m, 08.v.2009 (*R. Nattier*) (MNHN-CAELIF929-931); 1 juvenile, Grande Terre, Réserve de la Forêt Nord, Yaté (22°18’59.2’ S, 166°55’14.4’’E), 438 m, 08.v.2009 (*R. Nattier*) (MNHN-CAELIF932); 1♂, Grande Terre, Réserve naturelle intégrale de la Rivière Bleue, Haute Rivière Bleue, 5.iii.1986 (*J. Boudinot*) (MNHN-CAELIF933).

*Etymology.* The species is dedicated to the late Christiane Amédégnato, acridologist at MNHN, for her kind help at the beginning of this study and in memory of her outstanding contribution to acridid taxonomy.

*Distribution.* New Caledonia, Grande Terre, Province Sud: reserve of Forêt Nord, near the Goro Nickel factory, and Parc de la Rivière Bleue.

## Supporting Information

Figure S1 **Topologies obtained in Parsimony (a) and Bayesian inference (b) for all data sets.**
(TIF)

Figure S2 **Topologies obtained in separate analyses.** EF1a (a: Parsimony, b: Bayesian inference); Cytb (c: Parsimony, d: Bayesian inference); CO2 (e: Parsimony, f: Bayesian inference); H3 (g: Parsimony, h: Bayesian inference).
(PDF)

Table S1 **Molecular sampling of data discarded from phylogenetic analyses (GBK accession numbers).**
(PDF)
