# Supplementary figures and images for: Climate and Soil Type Together Explain the Distribution of Microendemic Species in a Biodiversity Hotspot
Source: PLoS One. 2013 Dec 18;8(12):e80811. doi: 10.1371/journal.pone.0080811 (PMC3867321; doi:10.1371/journal.pone.0080811)

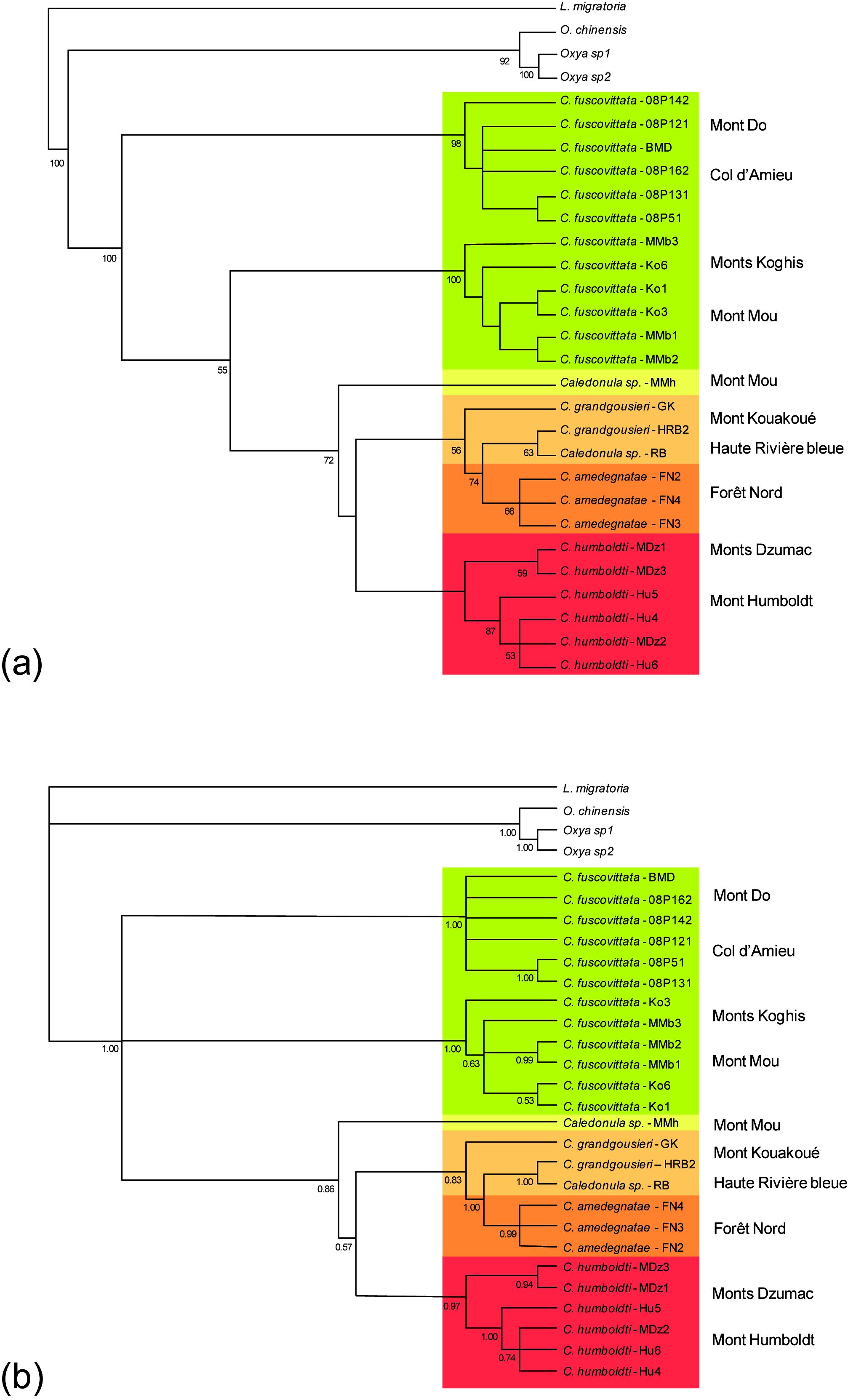

Supplement: Figure S1 — Topologies obtained in Parsimony (a) and Bayesian inference (b) for all data sets. (TIF) [file pone.0080811.s001.tif]

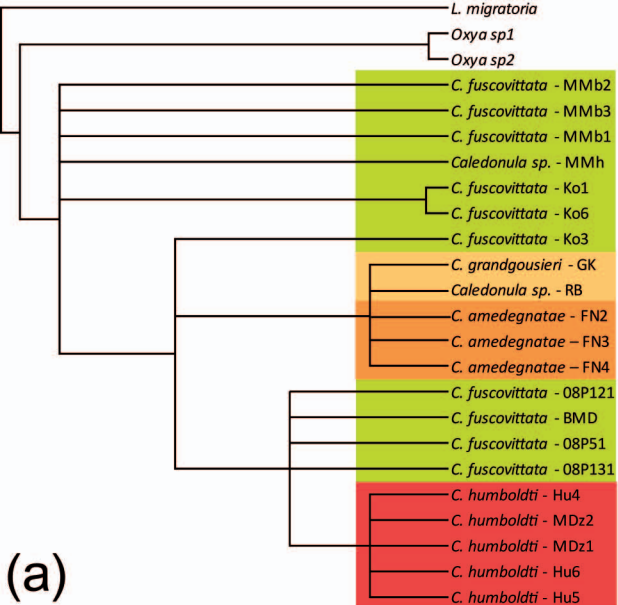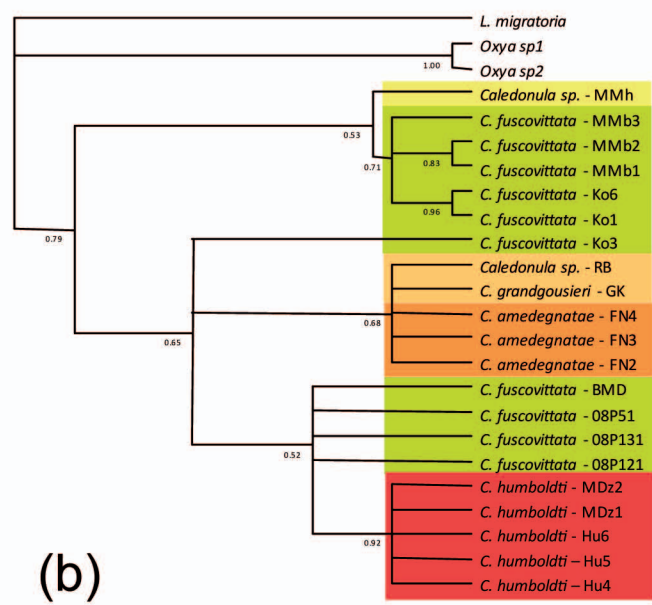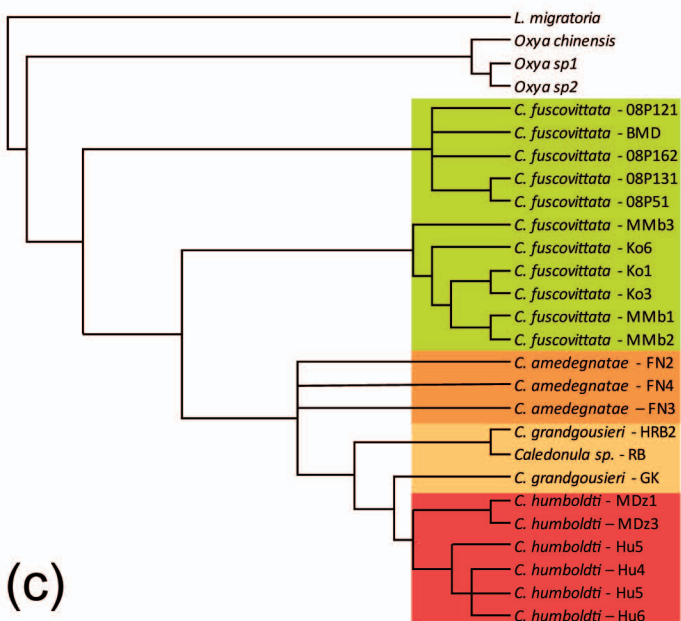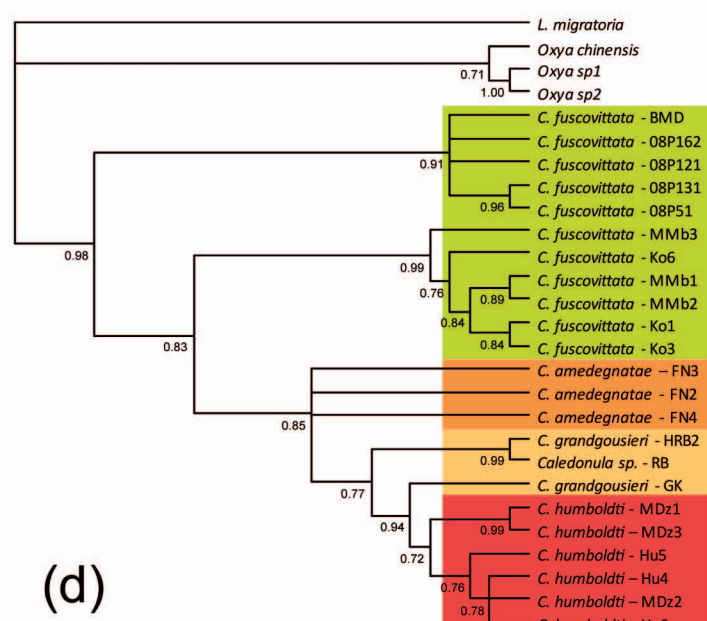

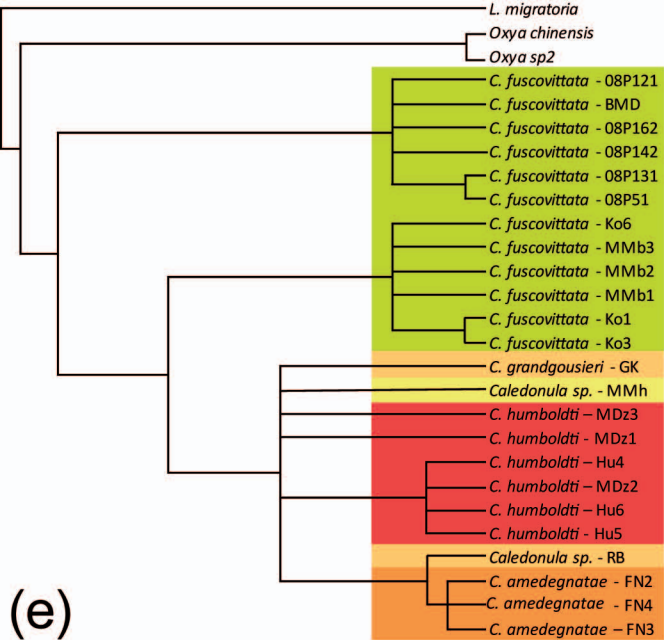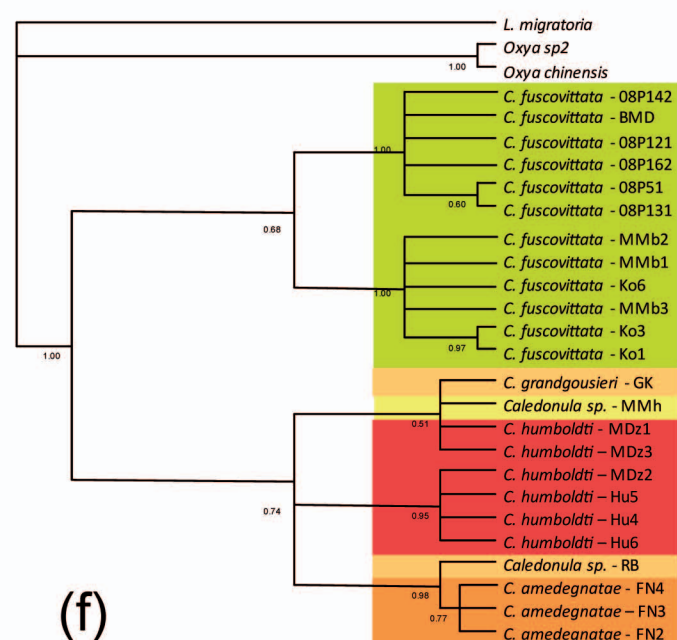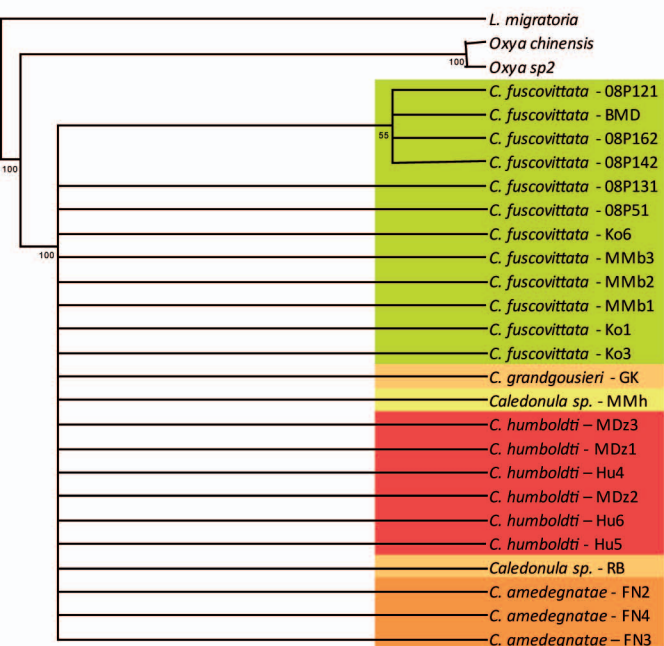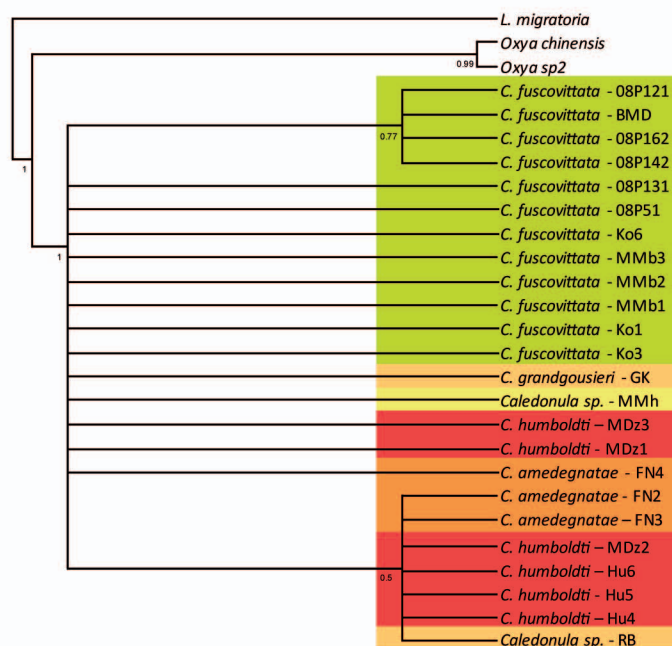

Supplement: Figure S2 — Topologies obtained in separate analyses. EF1a (a: Parsimony, b: Bayesian inference); Cytb (c: Parsimony, d: Bayesian inference); CO2 (e: Parsimony, f: Bayesian inference); H3 (g: Parsimony, h: Bayesian inference). (PDF) [file pone.0080811.s002.pdf]
