# Supplementary material for: Climate and Soil Type Together Explain the Distribution of Microendemic Species in a Biodiversity Hotspot
Source: PLoS One. 2013 Dec 18;8(12):e80811. doi: 10.1371/journal.pone.0080811 (PMC3867321; doi:10.1371/journal.pone.0080811)
Supplement: Table S1 — Molecular sampling of data discarded from phylogenetic analyses (GBK accession numbers). (PDF) [file pone.0080811.s003.pdf]

			Voucher specimen	GenBank accession number		
**Species**	**Locality**	**Sample code**	**MNHN collection number**	**CO1**	**ITS-1**	**28S**
C. fuscovittata	Mts Koghis	Ko1	MNHN-CAELIF903	KF772298	KF772415	-
		Ko3	MNHN-CAELIF904	KF772299	KF772416	KF772294
		Ko6	MNHN-CAELIF905	KF772300	KF772417	-
	Col d'Amieu	08P121	MNHN-CAELIF908	-	KF772412	-
		08P162	MNHN-CAELIF910	-	KF772414	-
		08P51	MNHN-CAELIF907	KF772297	-	-
		08P131	MNHN-CAELIF909	KF772296	KF772413	-
		08P142	MNHN-CAELIF906	-	-	-
	Mt Mou - bas	MMb1	MNHN-CAELIF900	KF772301	KF772418	-
		MMb2	MNHN-CAELIF901	KF772302	KF772419	-
		MMb3	MNHN-CAELIF902	KF772303	-	-
	Base Mont Do	BMD	MNHN-CAELIF911	-	-	-
C. humboldti n. sp.	Mts Dzumac	MDz1	MNHN-CAELIF924	KF772310	-	-
		MDz2	MNHN-CAELIF925	KF772311	-	-
		MDz3	MNHN-CAELIF934	-	-	-
	Mt Humboldt	Hu4	MNHN-CAELIF915	-	KF772422	-
		Hu5	MNHN-CAELIF916	KF772308	-	KF772295
		Hu6	MNHN-CAELIF919	KF772309	-	-
C. amedegnatae n. sp.	Foret Nord	FN2	MNHN-CAELIF928	KF772304	KF772420	-
		FN3	MNHN-CAELIF929	KF772305	KF772421	-
		FN4	MNHN-CAELIF933	KF772306	-	-
C. grandgousieri n. sp.	Mont Kouakoué	GK	MNHN-CAELIF913	KF772307	-	-
	Haute-Rivière bleue	HRB2	MNHN-CAELIF912	-	-	-
Caledonula sp. (juvenile)	Haut Mt Mou	MMh	MNHN-CAELIF935	KF772312	-	-
Caledonula sp. (juvenile)	Haute-Rivière bleue	RB	MNHN-CAELIF936	KF772313	-	-
Oxyinae sp1	-	Oxsp1	-	KF772314	-	-
Oxyinae sp2	-	Oxsp2	-	KF772315	-	-
Oxya chinensis	-	-	-	NC_010219	-	EF685940
Locusta migratoria	-	-	-	NC_001712	-	EF685941
